# Broadband Dielectric Spectroscopy as a Potential Label-Free Method to Rapidly Verify Ultraviolet-C Radiation Disinfection

**DOI:** 10.6028/jres.126.022

**Published:** 2021-08-20

**Authors:** Yaw S. Obeng, Brian J. Nablo, Darwin R. Reyes, Dianne L. Poster, Michael T. Postek

**Affiliations:** 1 National Institute of Standards and Technology, Gaithersburg, MD 20899, USA; 2 Vitreous Research Solutions, Rockville, MD 20851, USA; 3 University of South Florida, Tampa, FL 33612, USA

**Keywords:** biological indicators, disinfection, DNA, electrical properties, spectroscopy, thin films, ultraviolet-C

## Abstract

Microwave (MW) sensing offers noninvasive, real-time detection of the electromagnetic properties of biological materials via the highly concentrated electromagnetic fields, for which advantages include wide bandwidth, small size, and cost-effective fabrication. In this paper, we present the application of MW broadband dielectric spectroscopy (BDS) coupled to a fabricated biological thin film for evaluating ultraviolet-C (UV-C) exposure effects. The BDS thin film technique could be deployed as a biological indicator for assessing whole-room UV-C surface disinfection. The disinfection process is monitored by BDS as changes in the electrical properties of surface-confined biological thin films photodegraded with UV-C radiation. Fetal bovine serum (FBS, a surrogate for protein) and bacteriophage lambda double-stranded deoxyribonucleic acid (dsDNA) were continuously monitored with BDS during UV-C radiation exposure. The electrical resistance of FBS films yielded promising yet imprecise readings, whereas the resistance of dsDNA films discernibly decreased with UV-C exposure. The observations are consistent with the expected photo-oxidation and photodecomposition of protein and DNA. While further research is needed to characterize these measurements, this study presents the first application of BDS to evaluate the electrical properties of solid-state biological thin films. This technique shows promise toward the development of a test method and a standard biological test to determine the efficacy of UV-C disinfection. Such a test with biological indicators could easily be applied to hospital rooms between patient occupancy for a multipoint evaluation to determine if a room meets a disinfection threshold set for new patients.

## 1 Introduction

Many microorganisms (*e.g.*, bacteria, viruses, and fungi) are inactivated by exposure to ultraviolet-C radiation (UV-C, wavelengths 200 nm to 280 nm) through photo-induced transformations to deoxyribonucleic acid (DNA) and ribonucleic acid (RNA) [1]. These transformations disrupt the microorganisms’ processes of transcription, translation, replication, and reverse transcription [2-4]. These disruptions provide an effective approach towards inactivating biological activity. The germicidal action of UV-C requires a relatively close proximity and a direct line-of-sight to the target in order to achieve the appropriate inactivation dose. Despite these limitations, UV-C is quickly becoming a disinfection technology for whole-room disinfection in hospitals [2].

Traditional assessments of whole-room UV-C decontamination include visual inspection, bioluminescence adenosine triphosphate (ATP) analyses, and microbiological culturing. Visual inspections are often highly variable and unreliable. ATP and microbiological analyses require analytical laboratory facilities to process samples, which in turn delays the verification of sterilization and room turnover. Germicidal efficacy is frequently confirmed with basic microbiological techniques, where the surviving population is quantified by the number of colony-forming units (CFUs) from growth plate counts. Although the CFU-counting process is well established, the incubation time (*i.e.*, time to result) is on the order of 1 d to 2 d [3]. Effective quality-control measures for rapidly evaluating UV-C disinfection of whole rooms are limited due, in part, to the lack of tools for real-time feedback [2]. Quality-control programs for other sterilization processes (*e.g.*, steam sterilization) often include multiple components to monitor the sterilization, such as (1) biological indicators to verify biological inactivation (*e.g.*, *Geobacillus stearothermophilus*), (2) mechanical indicators (*e.g.*, gauges) that monitor the sterilization process, and (3) chemical indicators that undergo a visible change when a sterilization parameter has been met (*e.g.*, autoclave steam indicator tape). In the case of whole-room UV decontamination, disposable photodosimeters composed of UV-selective inks have emerged as promising candidates for spot checking the UV fluence for rapid UV-C disinfection inspection [5]. Their mechanism of photoconversion, however, is not a direct indicator of biological damage. Currently, a safe, noninfectious biological indicator is not available for rapidly verifying UV decontamination in hospital rooms.

A method for the rapid verification of photo-induced biological inactivation is urgently needed for whole-room UV-C disinfection processes. Ideally, this technology would monitor disinfection through a measurand that is correlated to biological activity, dispersible throughout a room for multipoint verification, and capable of real-time verification to support the high turnover in hospital environments. Furthermore, the measurement technology should be independent of the substrate and relatable to a broad spectrum of pathogens [6].

Broadband dielectric spectroscopy (BDS) is a nondestructive, noninvasive, low-power analytical methodology based upon the polarization of molecules in electromagnetic fields commonly evaluated in the frequency range of 0.3 GHz to 300 GHz, and it can differentiate between material compositions, including biological materials [7]. We applied this approach for the first time to solid-state biological thin films to show the potential of the coupled BDS–thin film technology for assessing UV disinfection. From the observations reported in this paper, BDS can detect electrical changes in biological thin films exposed to UV-C radiation. This prototype demonstration shows the potential of the BDS thin film technique to be developed as a rapid biological indicator for assessing the efficacy of UV-C disinfection. However, further development is required to fully realize the BDS thin film technique as a complementary analytical method for quality-control measurements of UV-C whole-room disinfection.

The precedent for BDS as a biological measurement relates to the bulk of research demonstrating the frequency-dependent permittivity and conductivity of analytes. When the analyte is a biological cell, dielectric measurements yield information about the state of cellular contents [8]. Biological tissues are dependent on several contributing factors (*e.g.*, water content, nucleus/cytoplasm ratio, and the concentrations of relevant ions, proteins, and other macromolecules [9]) that influence their electrical properties (*e.g.*, conductivity and permittivity). At microwave (MW) frequencies, the plasma membrane is transparent, which allows for direct probing of the cell’s contents. The electrical properties of cells can vary widely between normal and pathological states [10]. In principle, both dielectric and conducting analytes contribute to energy loss. The loss tangent of biological analytes, however, must be sufficiently large for the dielectric loss to exceed the conductor loss. For most cells, the total decline in permittivity (Δɛ) is linearly correlated with the volume fraction of pristine cells present [11, 12]. Typically, the radiofrequency (RF) dielectric properties of microbial suspensions are a direct and monotonic function of the volume fraction [11, 12], but other models have been developed [9, 13]. A review of the interaction of RF fields with biological systems (*i.e.*, the electrical properties of tissues, macromolecular solutions, and cell membranes) is also available [11, 14].

During the photodegradation process, the biological thin films will develop UV-altered species that possess different electrical properties than the pristine material. The total direct current resistance of the sample, calculated from the measured *S* parameters, is a concentration-weighted sum of resistance from the generated UV-altered species, the remaining pristine material, and any UV-inactive species (*e.g.*, ions and trace water). Harris *et al.* [15] demonstrated that the MW dielectric properties of biological analytes (*e.g.*, cell suspensions) are a direct monotonic function of the volume fraction of the analyte, differing significantly from other particulate matter in aqueous solutions. For most cells, the total dielectric constant of solutions declines linearly in proportion with the volume fraction (P) of intact cells up to the limits of 10% to 20% [8, 15]. MW signal loss in heterogeneous thin films, however, is more complicated and exhibits nonideal behavior because of the additional collective loss modes and small amounts of water content [15, 16]. Thus, the observed dielectric loss and the derived electrical resistance in thin films of biological samples represent the “effective” resistance of the entire film and may not be specific to a single species. Therefore, when the denatured species yields a lower electrical resistance than the pristine target material, the observed electrical resistance approaches a minimum as UV-C degradation of the population proceeds.

The novelty of the current work lies in the application of BDS to thin films of biological materials rather than solvated analytes, which affords a convenient “solid-state” test configuration for real-time on-site feedback [17]. Other prior MW dielectric studies of cells used analytes trapped inside the cavities of specialized waveguides, or analytes embedded in nanostructured matrices [18], which are complex and labor-intensive structures. We envision BDS technology as a rapid, sensitive, and simple detection method for monitoring the UV-C degradation of a biological indicator to assess the efficacy of UV-C germicidal equipment and whole-room UV-C disinfection protocols. We utilized unpatterned substrates of desiccated biological materials for our device under test (DUT) to minimize the cost and complexity of implementation. These thin films can be easily distributed throughout a whole room to monitor UV-C radiation efficacy across the environment and to avoid the introduction of additional biological hazards. We demonstrate that BDS can detect UV-C–induced changes in the electrical resistance of these biological thin films, in accordance with previously published BDS studies [11, 19]. This study, to the best of our knowledge, represents the first attempt to couple BDS with solid-state biological thin films. Further studies are required to quantify and correlate the analytical metrics to the biological viability of the most UV-C resilient microorganisms responsible for hospital-acquired infections.

## 2 Background Theory of BDS

MWs are electromagnetic radiation with wavelengths ranging from 1 mm to 1 m in free space and frequencies ranging from 300 MHz to 300 GHz. MWs are transmitted, absorbed, or reflected when they interact with a material, interrogating the entire analyte volume but not limited by form factor nor shape. This makes MWs very versatile for analytical chemistry applications [7]. Furthermore, MW-matter interactions are sensitive to chemo-electrical composition, including both intra- and intermolecular interactions. MWs lose energy when they interact with nonmagnetic materials through two predominant mechanisms: dielectric (dipolar) and conduction losses [10, 20]. When high-frequency MWs penetrate and propagate through a dielectric material, energy losses are mostly due to the polarization of dipoles attempting to align with the rotating field. The impact of the changing electric field on the polarization of the dipoles depends on the intrinsic properties (*e.g.*, polarizability and stereochemistry) of the molecules involved. At sufficiently high frequencies, the dipoles cannot reorient rapidly enough to the oscillating electric field, leading to dielectric loss.

MWs also induce translational motions of electrons, ions, or complex dipoles. In dielectric materials, however, MW absorption is largely dominated by dielectric repolarization. In principle, both dielectric properties and electrolytes contribute to the energy loss in biological samples. For dry thin films of FBS and dsDNA, we attribute the insertion losses detected by BDS to the changes in the dielectric properties caused by UV-C photodegradation.

In the following, we briefly examine the theoretical underpinnings of the BDS technique for establishing the experimental protocols and data analyses. The absorption of MWs is related to the material’s complex permittivity:

ɛ = ɛ_0_(ɛ' - jɛ"), (1)

where $\varepsilon_{0}$ is the permittivity in free space, ɛ' is the real part of the dielectric constant, and the imaginary part, ɛ", is the effective dielectric loss factor.

Here, we assume a uniform transmission line model such that the differential equations for the line voltage (V) and current (I) can be expressed in the frequency domain as [21]:

$\frac{dV}{dx}=-ZI,$ (2)

and

$\frac{dI}{dx}=-YV,$ (3)

where Z=R+jωL, and Y=G+jωC, where ω is as the angular frequency, and R, L, C, and G are the frequency-dependent line resistance, inductance, capacitance, and dielectric conductance per unit length, respectively. The solutions to Eq (1) and Eq. (2) can be expressed as:

$V=V_{A}e^{-\Gamma x}+V_{B}e^{\Gamma x},$ (4)

and

$I=I_{A}e^{-\Gamma x}+I_{B}e^{\Gamma x},$ (5)

where Γ is the propagation constant, and it is defined as [19]:

$\Gamma =\sqrt{ZY}=\sqrt{\left(R+j\omega L\right)\left(G+j\omega C\right)}$. (6)

The propagation constant Γ can also be represented electrically as [19]:


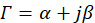
, (7)

where

$\alpha =\frac{R}{2\sqrt{L/C}}+\frac{G}{2}\sqrt{\frac{L}{C}}\;,$ (8)

and


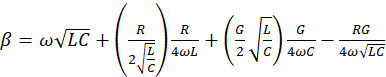


. (9)

We can further resolve the real part of the propagation constant, the attenuation constant (α), into dielectric and conductor losses:

$\alpha =\alpha_{c}+\alpha_{i}$, (10)

where


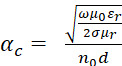
, (11)

and


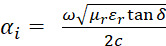
, (12)

and *µ*_0_ is the permeability of free space, *µ_r_* is the relative permeability, $n_{0}$ is the characteristic impedance of free space (377 Ω), $\varepsilon_{r}$ is the relative permittivity (*i.e.*, dielectric constant), σ is the conductivity of the metal, *d* is the separation distance between the signal line and its return path (dielectric thickness), *c* is the speed of light *in vacuo* (300,000 km/s), and tan⁡δ is the loss tangent of the dielectric in relation to the ratio of energy lost to energy stored per cycle,($i.e.,\tan\delta =\varepsilon_{r}^{''}/\varepsilon_{r}^{'})$. For completeness, we note that Eq. (8) and Eq. (9) are approximations for the low-loss case and are not exact for all loss cases [22].

**Fig. 1:**
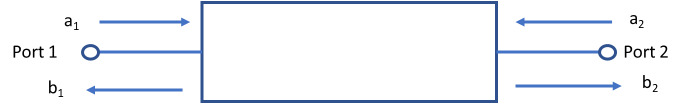
A schematic representation of a two-port VNA, as shown in Fig. 2, where:

When MWs interact with an analyte (*i.e.*, DUT), the signal scatters. A portion of the radiation is reflected backwards toward the source; the remaining signal is partitioned between absorption by the analyte and transmission to the receiver (Fig. 1). In the simple case where there is no or minimal net signal absorption as the signal travels through the analyte, the ratio of transmitted to reflected energies (*i.e.*, the extent of scattering) depends in part on the impedance mismatch between the DUT and the source; this is typically 50 Ω for most RF/MW applications. The scattering from the various electrical interfaces is summarized as a matrix that quantifies how RF energy propagates through a multiport network, such as a vector network analyzer (VNA). A typical two-port measurement contains four *S* parameters (*S*_11_, *S*_21_, *S*_12_, and *S*_22_) that are vector quantities representing the magnitude and the phase of the frequency-dependent characteristics of the analyte. Identical MW signals emanate from both ports 1 and 2 respectively and travel toward the opposite port. A portion of the incident wave exits through port 1 and is transmitted through the DUT and received at port 2 (dubbed *S*_12_), with a coefficient a_2_. A portion of the incident wave is also reflected (*S*_11_), with a coefficient b_1_. Similar *S* parameters, *S*_21_ and *S*_22_, are associated with port 2. The portion of the transmitted signal that exits the DUT exits with a different magnitude and phase from the incident signal. Thus, *S*_21_ and *S*_12_ also describe the phase difference in degrees between a transmitted signal and an incident signal. The *S* parameters can be analytically transformed to produce the characteristic circuit element of the analyte [21].

a_1_ is the signal into port 1,

b_1_ is the signal out of port 1,

a_2_ is the signal into port 2, and

b_2_ is the signal out of port 2.

The *S* parameters for this conventional element are defined as follows:

b_1_ = a_1_*S*_11_ + a_2_*S*_12_, (13)

and

b_2_ = a_1_*S*_21_ + a_2_*S*_22_, (14)

where

*S*_11_ is the port 1 reflection coefficient: *S*_11_ = b_1_/a_1_, where a_2_ = 0;

*S*_22_ is the port 2 reflection coefficient: *s*_22_ = b_2_/a_2_, where a_1_ = 0;

*S*_21_ is the forward transmission coefficient: *S*_21_ = b_2_/a_1_, where a_2_ = 0; and

*S*_12_ is the reverse transmission coefficient: *S*_12_ = b_1_/a_2_, where a_1_ = 0.

In this work, we used these *S* parameters to evaluate the changes in the analyte’s chemo-electrical properties. The BDS rapidly interrogates a dynamic range of material characteristics, offering potential future capabilities to metrology for healthcare applications based on previous works [9, 19].

## 3 Application of BDS to Monitor Photodecontamination Processes

Predicated on the idea of a biological indicator, we utilized BDS to monitor the UV degradation of thin films composed of biological material. Our proxy for protein was FBS (40 mg/mL protein, 30-2021, ATCC, Manassas, VA, USA),^1^1 Certain commercial equipment, instruments, or materials are identified in this report to specify the experimental procedure adequately. Such identification does not imply recommendation or endorsement by the National Institute of Standards and Technology, nor does it imply that the materials or equipment identified are necessarily the best available for the purpose. which is the serum portion of blood largely composed of protein and an appreciable number of ions and small molecules. Our DNA reference was purified dsDNA from bacteriophage lambda suspended in a pH 8 Tris Acetate-EDTA buffer (i.e., TE buffer) (500 µg/mL, N3011S, New England Biolabs, Ipswich, MA, USA). These biological agents were used as received from the manufacturer. Thin films (*n* ≥ 3) were generated by spreading 75 µL of the stock across 75% of the area of a glass coverslip (18 mm^2^, no. 1 1/2, Corning, Corning, NY) precleaned with sonication in deionized (DI) water and subsequent 60 s exposure to oxygen plasma. The films were allowed to dehydrate in a biosafety hood for 2 h and then transferred to a vacuum desiccator for an additional 2 h of dehydration. The resulting surface densities were 2.8 µg/mm^2^ of DNA for dsDNA films and 0.2 mg/mm^2^ of protein for FBS films. This method is an inexpensive means of generating numerous biological indicators for run-to-run evaluation of UV-C disinfection throughout an entire room.

**Fig. 2:**
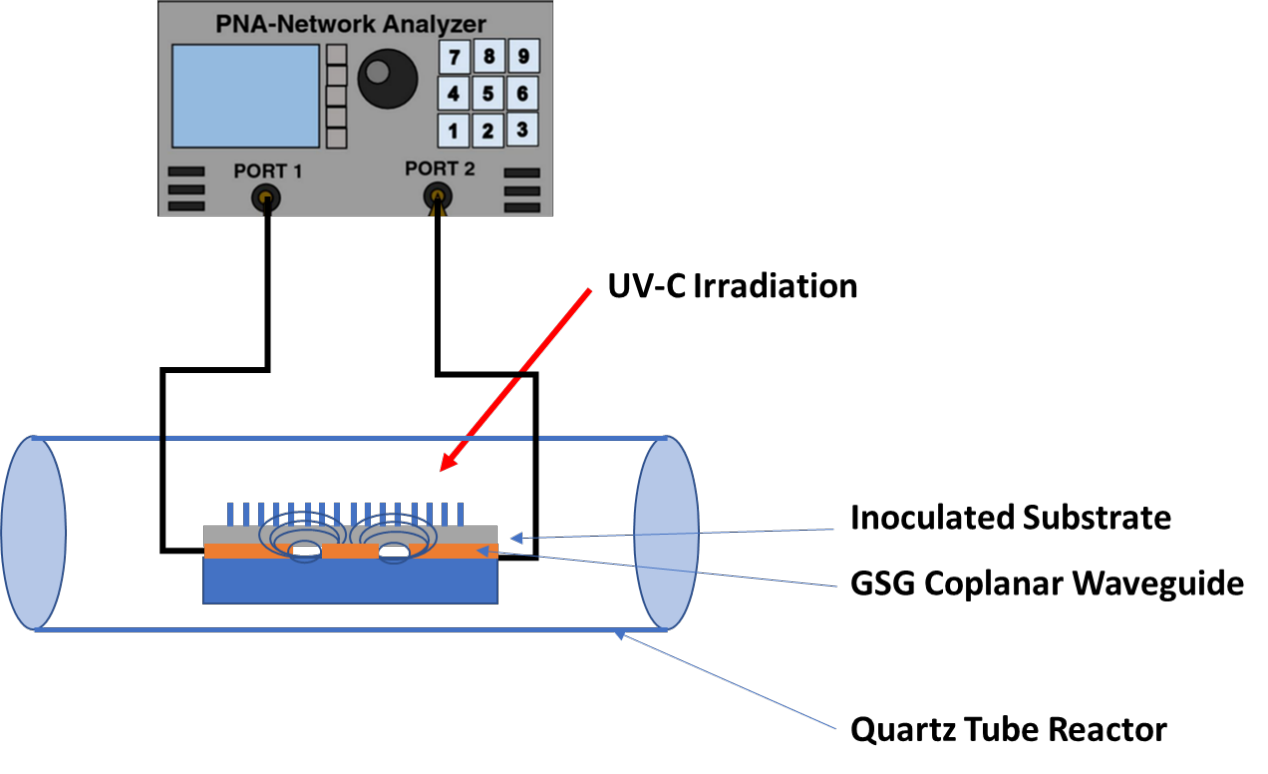
Schematic representation of the experimental setup with the inoculated substrate on a ground-signal-ground (GSG) waveguide situated in a controlled environment (quartz tube).

Figure 2 shows a schematic representation of the experimental setup. The reactor is composed of a ground-signal-ground (GSG) coplanar waveguide (CPW) situated in a controlled environment (quartz tube). The CPW was fabricated from a tin-covered printed circuit board (FR4), 61 mm long, with 5 mm wide ground lines separated from the 1.5 mm wide signal line by 1.2 mm gaps. The cables from the VNA were connected to the CPW with edge mount connectors (SMA connectors, Amphenol RF, Danbury, CT). The cables connecting the CPW were de-embedded, with a two-port short-open-load-through (SOLT) calibration in which the calibration standards were attached to the end of the feed cables (*i.e.*, the reference plane of the measurement was moved from the port faces of the VNA to the connector/cable interface). Thus, the launch connectors and CPW were part of the DUT. In this configuration, the reported *S* parameters are produced from the inoculated substrates perturbing the electric fields emanating from the signal and terminating on the ground lines in the CPW. Although we monitored a broadband range (0.1 GHz to 20 GHz), the reporting frequency of 1.1 GHz was chosen based on the sample dimensions, the absence of resonances, and minimized signal reflections.

After initial readings, the samples were illuminated with an UV-C lamp (Analytik Jena 95-0016-14 Shortwave UV lamp, 4 W, 115 VAC/60 Hz, Cole-Palmer, Vernon Hills, IL, USA) positioned 5 cm above the outer wall of the quartz reactor (3.8 cm diameter). BDS measurements were performed while the FBS and dsDNA films were continuously irradiated at a fluence rate of 100 µW/cm^2^ (measured at the reactor walls). The UV-C radiation fluence experienced by a DUT within our apparatus is 900 J/m^2^ for every 15 min of UV-C exposure. No attempt was made to culture microorganisms for comparisons with the thin films for this demonstration.

**Fig. 3:**
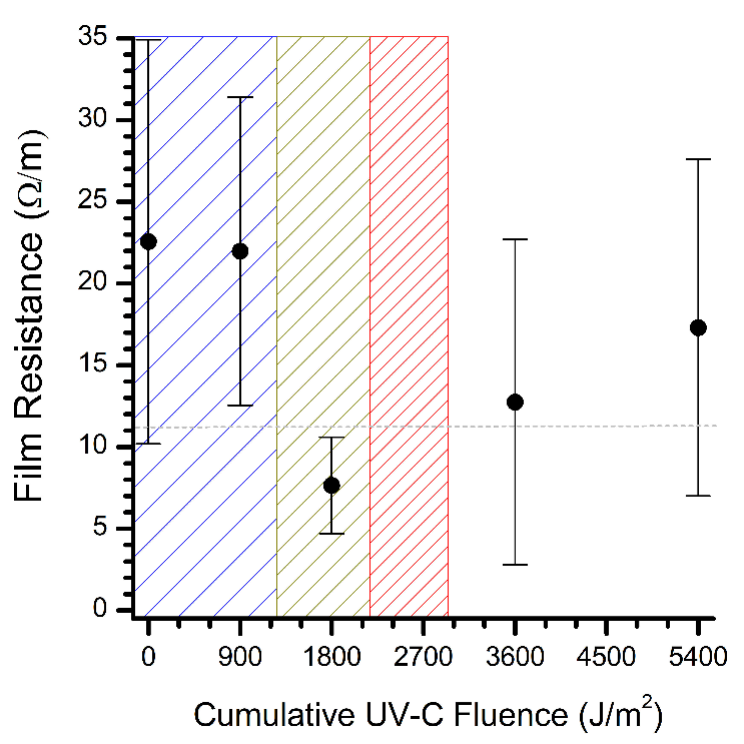
Evolution of electrical resistance of FBS thin films on glass substrates during UV-C exposure in air. The electrical resistance was calculated at a frequency of 1.1 GHz. The error bars represent the standard deviation of at least three samples. The dashed line represents the resistance of the blank substrate. A UV-C fluence of 900 J/m^2^ equates to 15 min of UV-C exposure within our apparatus. The leading edge of the blue, yellow, and red hashed zones represents the UV-C fluence required to inactivate UV-resilient *Aspergillus niger* spores by a 1 log_10_, 2 log_10_, and 3 log_10_ units reduction, respectively [1, 26].^2^2 3 log_10_ units refers to a 99.9% reduction, calculated as log_10_ (*N*_0_/*N*), where *N*_0_ is the initial value, and *N* is the final value.

Figure 3 shows the evolution of the protein’s electrical resistance during UV-C exposure in open air. After an initial drop, the electrical resistance of FBS increased and yielded inconsistent electrical resistances with increasing UV-C fluence. The initial loss of film resistance with FBS is consistent with rapid photobleaching of aromatic residues [23]. For example, Hegedus *et al.* have shown that proteins are susceptible to photodamage because aromatic amino acids readily absorb UV-C radiation [24]. Continued exposure to UV-C radiation resulted in an increase in the film’s resistance, which we attributed to gradual photo-oxidation, whereby atmospheric oxygen and protein residues are excited and react with each other [25]. Although changes in the electrical resistance were observed for the protein sample, analysis of variance statistics could not determine any significant difference between contiguous readings. Certainly, more work is necessary to qualify protein as a biological indicator for monitoring UV-C degradation with BDS, perhaps with highly purified protein samples instead of whole serum.

**Fig. 4:**
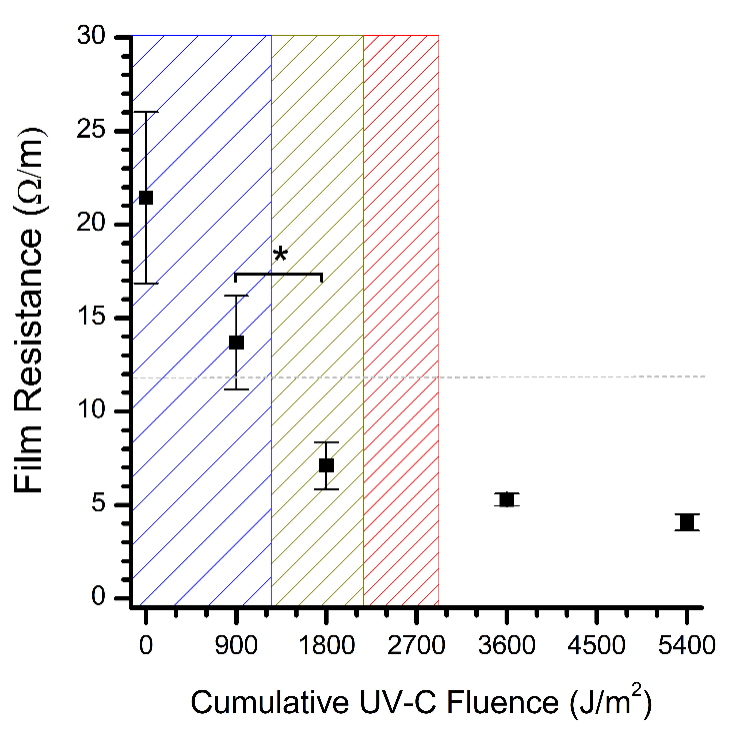
Evolution of electrical resistance of dsDNA thin films on glass substrates during UV-C radiation exposure in air. The electrical resistance was calculated at a frequency of 1.1 GHz. The error bars represent the standard deviation of at least three samples. The dashed line represents the resistance of the blank substrate. A UV fluence of 900 J/m^2^ equates to 15 min of UV exposure within our apparatus. The asterisk represents the significant difference between the bracketed points as determined by ANOVA (*P* value < 0.05). The leading edge of the blue, yellow, and red hashed zones represents the UV-C fluence required to inactivate UV-resilient *Aspergillus niger* spores by a 1 log_10_, 2 log_10_, and 3 log_10_ units reduction, respectively [1, 26].^2^

In contrast to the FBS readings, the electrical resistance of the dsDNA clearly decreased with increasing UV-C fluence (cf. Fig. 3 to Fig. 4). Thin films of dsDNA were expected to be an exceptional biological indicator for UV-C efficacy based on the demonstration of phage T7 as a biological UV-C dosimeter via DNA quantification [24]. Photodegradation of DNA is well understood and typically regarded as the dimerization of nucleobases into cyclobutane pyridimine dimers, which disrupt pi-stacking and associated charge transport between nucleobases [27]. Thus, an increase in the film’s electrical resistance would be expected as charge transport is further disrupted by continued UV photodegradation. As shown in Fig. 4, however, the resistance of the dsDNA thin films decreased with UV-C fluence, suggesting that BDS may be observing more than the nucleobases. After 30 min (1800 J/m^2^), an analysis of variance (ANOVA) verified a significant decrease in the detected electrical resistance of the dsDNA films, which was just beyond the critical UV-C fluence (1220 J/m^2^) required to induce a 1 log_10_ units reduction in the population of UV-resilient *Aspergillus niger* spores without photoreactivation [1, 26]. Coincidentally, the decline of the dsDNA thin film resistance in Fig. 4 is very reminiscent of the inactivation of severe acute respiratory syndrome coronavirus 2 (SARS-CoV-2) by UV-C radiation [28]. Although further research is required to determine the molecular events, our BDS measurements demonstrate that dsDNA films are a conceivable biological indicator for UV-C disinfection.

For the data presented, a UV-C fluence of 5400 J/m^2^ far exceeds that of a germicidal dose for UV-resistant microorganisms (*e.g.*, 2930 J/m^2^ for a 3 log_10_ units reduction of *Aspergillus niger* spores) [1, 26]. At these high doses, photo-oxidization will likely dominate, which is analogous to the removal of organic debris by UV ozone cleaners via the UV conversion of atmospheric oxygen into ozone (O_3_) and reactive atomic oxygen (O*) [29, 30]. Nevertheless, the observed changes in the electrical resistance of the dsDNA thin films by UV-C radiation provide a demonstration of the application of BDS to thin films to monitor UV-C disinfection. More work is essential to establish linkages between the BDS measurements and DNA photo-induced damage, such as quantitating the amount of DNA damage with quantitative polymerase chain reaction. Results could be used to model and predict the attributes that are necessary to fabricate robust dsDNA solvent-free thin films for use as biological indicators for BDS monitoring of UV-disinfection processes [31].

## 4 Prospective Application of BDS as a UV Sterilization Metrology Standard

The observable UV-induced changes in FBS and dsDNA thin films warrant the examination of the BDS technique as a potential tool to be used with noninfectious biological indicators to support quality-control measurements for whole-room UV-C disinfection [32]. Further research is needed to correlate the response of BDS thin film technology to the UV-C fluence necessary to inactivate UV-resilient pathogens, such as *Aspergillus niger* spores. The BDS technology described herein underpins the UV-C antimicrobial metrology standards being developed through a collaborative effort involving the National Institute of Standards and Technology, the International Ultraviolet Association and its affiliates, the UV industry, and academic and public health stakeholders including the Yale School of Medicine [33].

## 5 Conclusions

In this paper, we demonstrated the capability of BDS to detect UV-induced changes in the electrical resistance of FBS and dsDNA thin films. The electrical resistance of dsDNA thin films demonstrates a greater precision and statistical reliability relative to FBS thin films. These BDS measurements suggest that dsDNA thin films could be a noninfectious biological indicator for UV-disinfection procedures. The simplicity and low cost of dsDNA films make them an attractive candidate for a biological indicator with the BDS technique. Their further development may provide a rapid spot check for confirming the efficacy of UV-C decontamination for whole-room disinfection in hospitals or other environments with high-touch biological-laden surfaces, such as corridors or elevators.
